# Diabetes knowledge, health literacy and diabetes self-care among older adults living with diabetes in Alexandria, Egypt

**DOI:** 10.1186/s12889-024-20238-w

**Published:** 2024-10-16

**Authors:** Soha Magdy Ahmed Abdallah, Abla Ibrahim Ayoub, Mohamed Mohei Eldin Makhlouf, Ayat Ashour

**Affiliations:** 1 Alexandria University Students hospital, Alexandria University, Alexandria, Egypt; 2 Department of Family Health, High Institute of Public Health, Alexandria University, Alexandria, Egypt

**Keywords:** Diabetes, Older adults, Health literacy, Self-care activities

## Abstract

**Background:**

Type 2 diabetes is a rising health problem, especially in older adults. Health literacy and the degree of diabetes knowledge are among the factors that may influence diabetes self-care activities. The aim of this study was to assess factors affecting self-care activities among older adults living with type 2 diabetes in Alexandria, Egypt.

**Methods:**

A cross-sectional study included 400 older adults over the age of 60 with type 2 diabetes, recruited from diabetes outpatient clinics affiliated to the health insurance organization in Alexandria, Egypt. A predesigned, structured interview questionnaire was used to assess sociodemographic factors, personal habits, medical history, and drug history. The All Aspects of Health Literacy Scale (AAHLS) and the numeracy section of the Short Test of Functional Health Literacy in Adults (STOFHLA) were used to assess the level of health literacy. Diabetes Knowledge Test 2 (DKT2) was used to assess diabetes knowledge and Summary of Diabetes Self-care Activities scale was used to assess self-care activities. Multiple logistic regression analysis was used to examine the relationship between health literacy and self-care.

**Results:**

The mean age of the participants was 65.75 ± 5.15 years, and 56.2% of them were males. The mean duration of diabetes was 10.61 ± 5.28 years, 14.3% were illiterate, and 37.2% were university graduates. Positive correlations were found between health literacy, diabetes knowledge, and diabetes self-care activities (*p* < 0.001). Health literacy and diabetes knowledge were found to be significant predictors of diabetes self-care activities in older adults (aOR = 1.132; 95% CI:1.062–1.207, *p* < 0.001 and aOR = 1.313; 95% CI: 1.178–1.464, *p* < 0.001; respectively).

**Conclusions:**

Health literacy and diabetes knowledge were found to be predictors of good self-care activities in older adults living with diabetes. Health educators and health care professionals should focus on health education and the enhancement of diabetes knowledge to improve self-care activities and eventually glycemic control in older adults living with diabetes.

## Contributions to the literature

- The study sheds light on health literacy as an important and relatively new health parameter that is not thoroughly studied among older adults in developing countries, particularly Egypt.
- Among older adults living with diabetes, knowledge of diabetes and health literacy were essential determinants of self-care behaviors.
- Educating people with diabetes and promoting health literacy can help seniors care for themselves and have better health outcomes.

## Background

Diabetes mellitus is a global public health problem that has been rising for the past few decades. In 2021, 537 million individuals between 20 and 79 years old had diabetes and it is predicted to reach 643 million by 2030 and 783 million by 2045. Among adults aged 75–79 years diabetes prevalence was estimated to be 24.0% in 2021 and predicted to rise to 24.7% in 2045. The aging of the world’s population will produce an increasing proportion of those with diabetes being over the age of 60 years [1].

The U.S. Department of Health and Human Services, Office of Disease Prevention & Health Promotion defined health literacy (HL) as “the degree to which individuals have the ability to find, understand, and use information and services to inform health-related decisions and actions for themselves and others” [2]. Personal, situational, and socio-environmental factors were found to be associated with HL. Personal factors include age, gender, education, income, occupation and health insurance. Situational factors include marital status, living situation and social support. Socio-environmental factors include culture and spoken local language [3]. Low HL has been reported as a significant predictor of poor health outcomes. Patients with low HL tend to be frequent users of emergency medical services [4]. Health Literacy was categorized into three levels; functional/basic literacy, which includes simple reading and writing skills and basic understanding of common diseases and health care systems; communicative literacy, which includes communication, social skills and critical literacy which includes enhanced cognitive skills that can be used to evaluate information and apply it to regulate situations which will affect the health of the individual and the entire community [5].

Diabetes specific HL skills include understanding verbal or written directions, apprehending appointment details, comprehending educational brochures and reading instructions or labels on pill bottles and fully understanding informed consent documents [6]. Patients with diabetes must know the signs and symptoms of hyperglycemia and hypoglycemia, how to correctly self-administer oral medications and insulin to achieve the best glycemic control. They must also know appropriate foot-care routine and how to monitor their blood glucose level and what to do if the levels are very high or very low [7].

Age was found to be negatively associated with diabetes knowledge and self-care practices, with older adults having less knowledge about managing their condition and its complications [8]. The Health and Retirement Study in the USA reported that impairments in self-care activities were more prevalent among older adults with diabetes compared to non-diabetic older adults [9].

Diabetes is an important chronic condition that requires extensive knowledge, continuous education and self-care management. Patients who are involved in their own disease management have a much better probability of learning about the disease and tend to maintain good glycemic control and adhere to self-care activities that could prevent complications [10]. Several studies reported that good diabetes knowledge had a strong positive association with better glycemic control and better HbA1c levels [11, 12].

Although diabetes self-management in Egypt was previously investigated [13]. Our research aims to address a specific gap by concentrating on older adults and examining the relationship between health literacy, diabetes knowledge, and self-care activities. By focusing on this vulnerable and underexplored population, our study seeks to generate insights that can inform targeted interventions and enhance diabetes outcomes among older adults in Egypt.

## Methods

### Study design, setting, and study participants

A cross-sectional study was conducted among 400 older adults with type 2 diabetes and extended over a three-month period between June and August 2021. Study participants were recruited from two outpatient clinics affiliated to health insurance organization in Alexandria, Egypt. These clinics were selected because they have the highest attendance rate of diabetic patients treated on an outpatient basis in Alexandria. We excluded older adults with any communication problems such as hearing impairment. Sample size was calculated based on the assumption that 50% of patients have insufficient information about diabetes. Using a margin error of 5%, and alpha error of 0.05, the minimum sample size required was 384 which was rounded to 400 participants. The sample size was calculated using Epi info7 software.

### Measures

A questionnaire was used to assess health literacy, diabetes literacy and self-care activities in older adults living with diabetes. The questionnaire was composed of the following parts:

1. **Predesigned structured interview questionnaire to collect the following data**: socio-demographic data (such as age, gender, marital status, education, income, occupation and living situation), personal habits such as practicing regular physical activities (participant was considered physically active if he or she achieved 30 min of exercise, 5 times per week), smoking status and diabetes history including duration of diabetes and current treatment.
2. **The Arabic version of All Aspects of Health Literacy Scale (AAHLS)** [14]
  The scale was originally developed by Chinn and McCarthy in 2013 as an effective measure of health literacy [15]. It is composed of 14 items assessing reading skills and understanding health information (Functional literacy), communication with health professionals (Communicative literacy) and using health information and capability to take action for one’s health (Critical literacy). AAHLS is a 3-point Likert scale scored as follow: “rarely” (0), “sometimes” (1), and “often” (2). Only the functional literacy section is scored as “rarely” (2), “sometimes” (1) and “often” (0). Higher scores indicate better health literacy levels.
3. **The Arabic version of Short Test of Functional Health literacy in Adults (STOFHLA)** [16]
  The original version of STOFHLA was created by Baker et al. [17]. The STOFHLA has two sections: reading section and numeracy section. Only the numeracy section was used in the present study. It consists of four questions that evaluate understanding of glucose monitoring, prescription labels and appointment notice. The original total score for the STOFHLA is 100 points, 70 points for the reading section and 30 points for the numeracy Sect. (7.5 points for each correct answer of the four questions). Higher scores in each section indicate better functional health literacy levels.
4. **The Arabic version of Summary of Diabetes Self-Care activities Scale (SDSCA)** [18]
  The original English version of SDSCA was developed by Toobert et al. in 2000 [19]. It consists of eight questions that assess four main aspects of diabetes self-care: diet, exercise, blood-glucose testing and foot care. The mean number of days per week for all four aspects of diabetes self-care activities (diet, exercise, blood glucose testing and foot-care) was calculated. Scores range from 0 to 7 with higher scores suggesting better self-care activities. We classified the responses into two categories according to the total mean score of SDSCA after another study done in 2016, with scores (˂ 3) representing poor self-care activities and (≥ 3) representing good self-care activities [20].
5. **The Arabic version of the revised brief Diabetes Knowledge Test (DKT2)** [21]
  The DKT2 was developed by Fitzgerald et al. in 2016 to assess the general knowledge of diabetes and its self-care [22]. DKT2 contains 23 questions and is divided into two parts that can be used separately. Part 1 was translated and validated in Arabic by Alhaiti et al. in 2016 [21]. It contains 14 questions that test the general knowledge about diabetes, and it is suitable for both type 1 and type 2 diabetes on any treatment. Part 2 contains 9 questions, and it is designed for patients who are using insulin only. Part 2 was translated by the research team using forward backward translation method. The total questionnaire consists of multiple-choice questions and each right answer was given one point. The higher the score is, the higher level of diabetes literacy the participant has. The original questionnaire does not have cut off scores for categorization of diabetes knowledge. A percent score (percent score = total score of each participant / total number of questions x 100) was calculated for all the participants, both who answered part 1 (14 questions) and those who answered both parts 1 and 2 (23 questions), so that all of the participants could be categorized together. The total percent score was categorized according to a study done in 2016 as; low diabetes knowledge (≤ 59%), average diabetes knowledge (60–74%) and high diabetes knowledge (≥ 75%) [20].
6. **Anthropometric measures**: Weight and height of each participant were measured to calculate the Body Mass Index (BMI). The weight in kilograms and the height in meters were obtained using a standard office scale and by the standard procedure. The body mass index was calculated using the following formula: BMI = kg/m^2^ [23]. Participants were categorized according to BMI into 3 categories according to the WHO classification for adults over 20 years old: Normal weight: 18.5–24.9 kg/m^2^, Overweight: 25–29.9 kg/m^2^ and Obese: ≥ 30 kg/m^2^.
7. **Blood pressure (BP) measurement**: The BP of each participant was carefully measured using a standard mercury device twice and the mean of the two measurements was taken. We classified blood pressure according to the American diabetes association (ADA) recommendations for target BP in older adults with diabetes into 3 categories as follows [24]: controlled BP (< 140/90), uncontrolled BP (≥ 140/90) and isolated systolic hypertension (systolic BP ≥ 140 + diastolic BP < 90).
8. **Glycated hemoglobin** (HbA1c in %) was obtained from participants’ records.

Prior to data collection, informed consent was obtained from each participant, wherein they were thoroughly informed about the purpose of the study, the type of data being collected, and their right to withdraw from the study at any point without any consequences. All personal identifiers were removed from the data to maintain anonymity. The data were coded and stored in a secure, password-protected database accessible only to the research team. Additionally, the study protocols were reviewed and approved by the institutional review board (IRB) to ensure compliance with ethical guidelines.

A pilot study (*n* = 20) was conducted to check the accuracy, reliability and the time needed to complete the questionnaire, there were no corrections needed. Time needed to complete the questionnaire ranged from 30 to 40 min. Participants in the pilot study were not included in the study sample. The clinics were visited 4 times per week. The average number of interviewed older adults per day was from 5 to 8. Response rate was 92%.

### Statistical methods

The data was managed and analyzed using statistical software SPSS version 25 (Armonk, NY: IBM Corp). The Kolmogorov-Smirnov test was used to assess if the data follow normal distribution. Our data was found to be not normally distributed. Qualitative data were described using frequency and percentage. Quantitative data were described using median and interquartile range. Spearman’s correlation coefficient was determined for linear correlation of two quantitative variables. Chi-squared (χ2) test was used to analyze the associations between qualitative data. Multivariate logistic regression was calculated to assess predictors of good diabetes self-care activities. All variables in the bivariate analysis with *p* value < 0.2 were entered into the model. The significance of the obtained results was judged at the 5% level.

## Results

The study sample included 400 older adults living with diabetes; the majority were males (56.2%). The age of the participants ranged between 60 and 87 years with a mean of 65.75 ± 5.15 years and 77% of them were in the youngest age group (60–69 years). More than half of the study sample were married (61.2%) and nearly two thirds were below university level (62.7%). 43% (43.2%) of the participants reported that they did not have enough income or were in debt. Most of them (88.5%) were not working (housewives or retired). Regarding living situation, only 16.2% were living alone (Table 1). The table also shows that only 22% of the study sample were practicing regular physical activities and only (9.3%) were current smokers. Nearly half (47.5%) had diabetes for 10 years or more, 65.5% were managed with oral hypoglycemic drugs and (16.0%) were managed with both oral hypoglycemic drugs and insulin. Regarding BMI, more than half of (51.2%) were overweight. According to ADA recommendations for target BP in older adults with diabetes, 23.8% had uncontrolled blood pressure and 13.2% had isolated systolic hypertension. Moreover, most of the sample had uncontrolled diabetes as measured by HbA1c (79.4%) (Table 1).

**Table 1:** General characteristics of the study participants

Variables	Older adults living with diabetes (*n* = 400)	
	No.	%
**Age (years)**		
60–64	202	50.5
65–69	106	26.5
70+	92	23.0
**Mean ± SD**	65.75 ± 5.15	
**Gender (**Male)	225	56.2
**Marital status (**Married)	245	61.2
**Education**		
Illiterate or Read & Write	57	14.3
Elementary	71	17.8
Preparatory	34	8.5
High school / technical diploma	89	22.2
University graduate or above	149	37.3
**Income (**Not enough / In debt)	173	43.2
**Occupation (**Not working [housewife, retired])	354	88.5
**Living condition**		
Alone	65	16.2
Family	335	83.8
**Practicing regular physical activities** (Yes)	88	22.0
**Current smoking** (Yes)	37	9.3
**Duration of diabetes (Years)** ≥ 10	190	47.5
**Mean ± SD**	10.61 ± 5.28	
**Diabetes medications**		
Oral hypoglycemic	262	65.5
Insulin	74	18.5
Both	64	16.0
**Body mass index (BMI kg/m2)**		
Normal weight (18.5–24.9)	54	13.5
Overweight (25–29.9)	205	51.2
Obese (≥ 30)	141	35.3
**Blood pressure**		
Controlled BP (< 140/90 mmHg)	252	63.0
Uncontrolled BP (≥ 140/90 mmHg)	95	23.8
Isolated Systolic hypertension (≥ 140 / <90 mmHg)	53	13.2
**HbA1c (***n*** = 340)** Uncontrolled (≥ 7%)	270	79.4

**Fig. 1:**
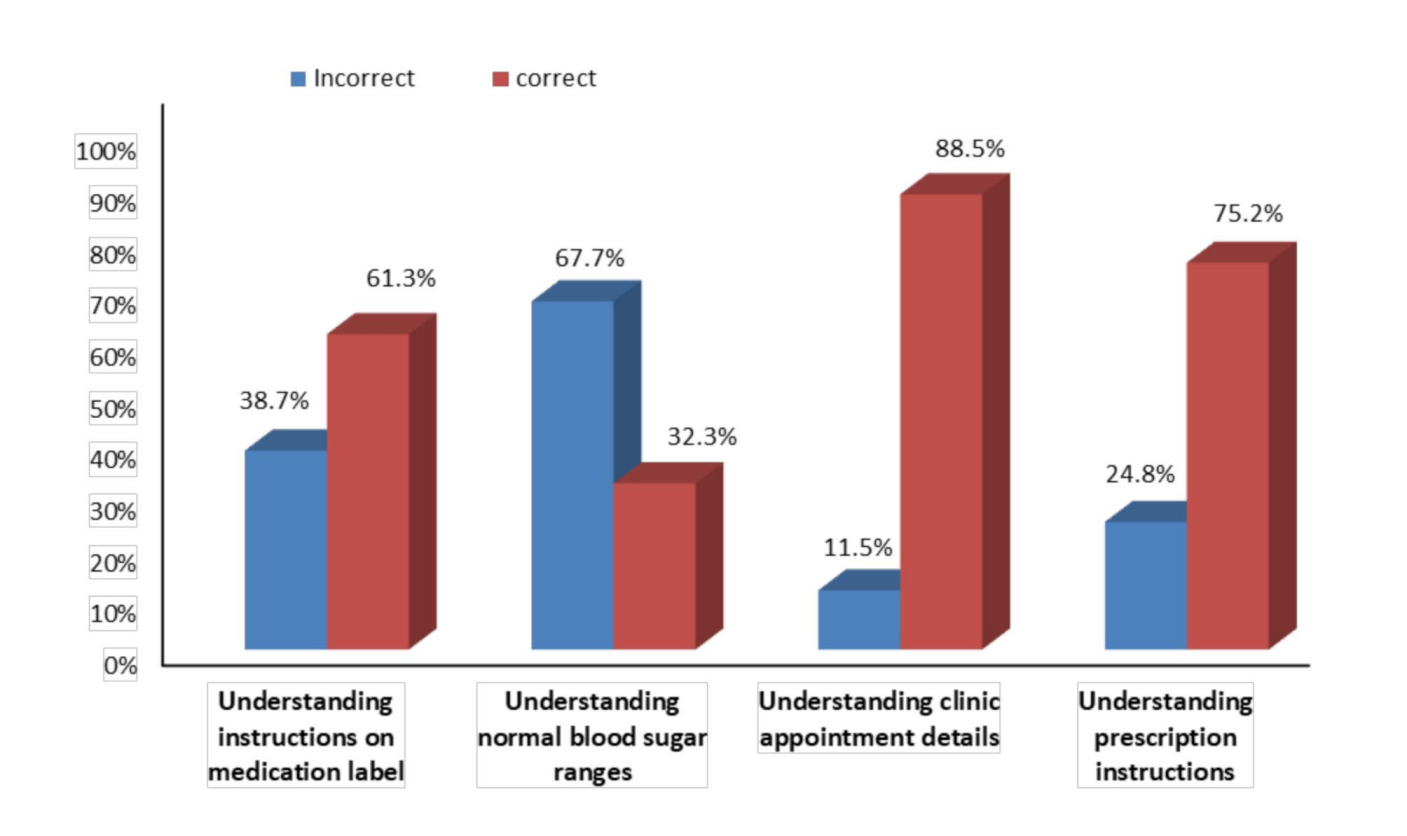
Categories of the numeracy section of the Short Test of Functional Health Literacy in Adults (STOFHLA)

**Fig. 2:**
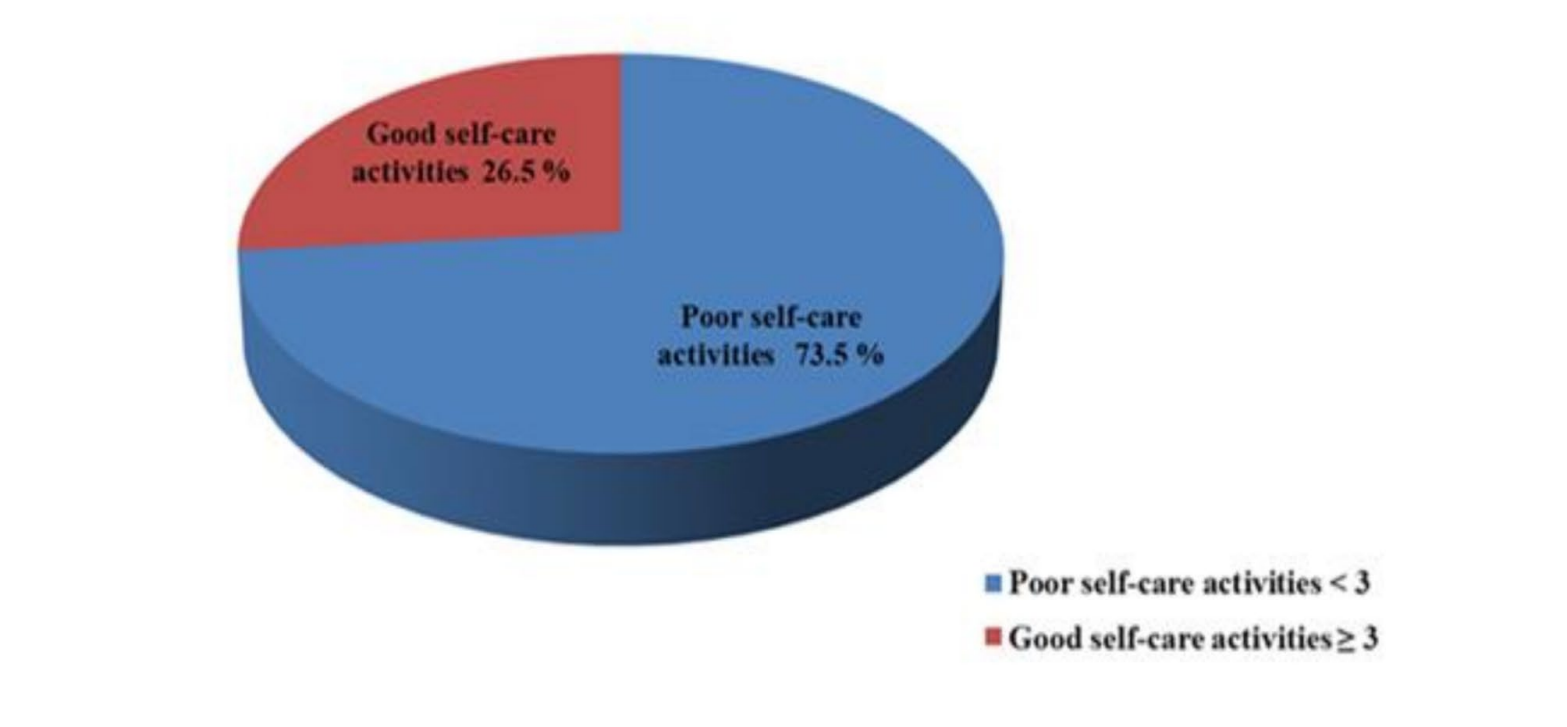
Self-care activities classification based on SDSCA

**Fig. 3:**
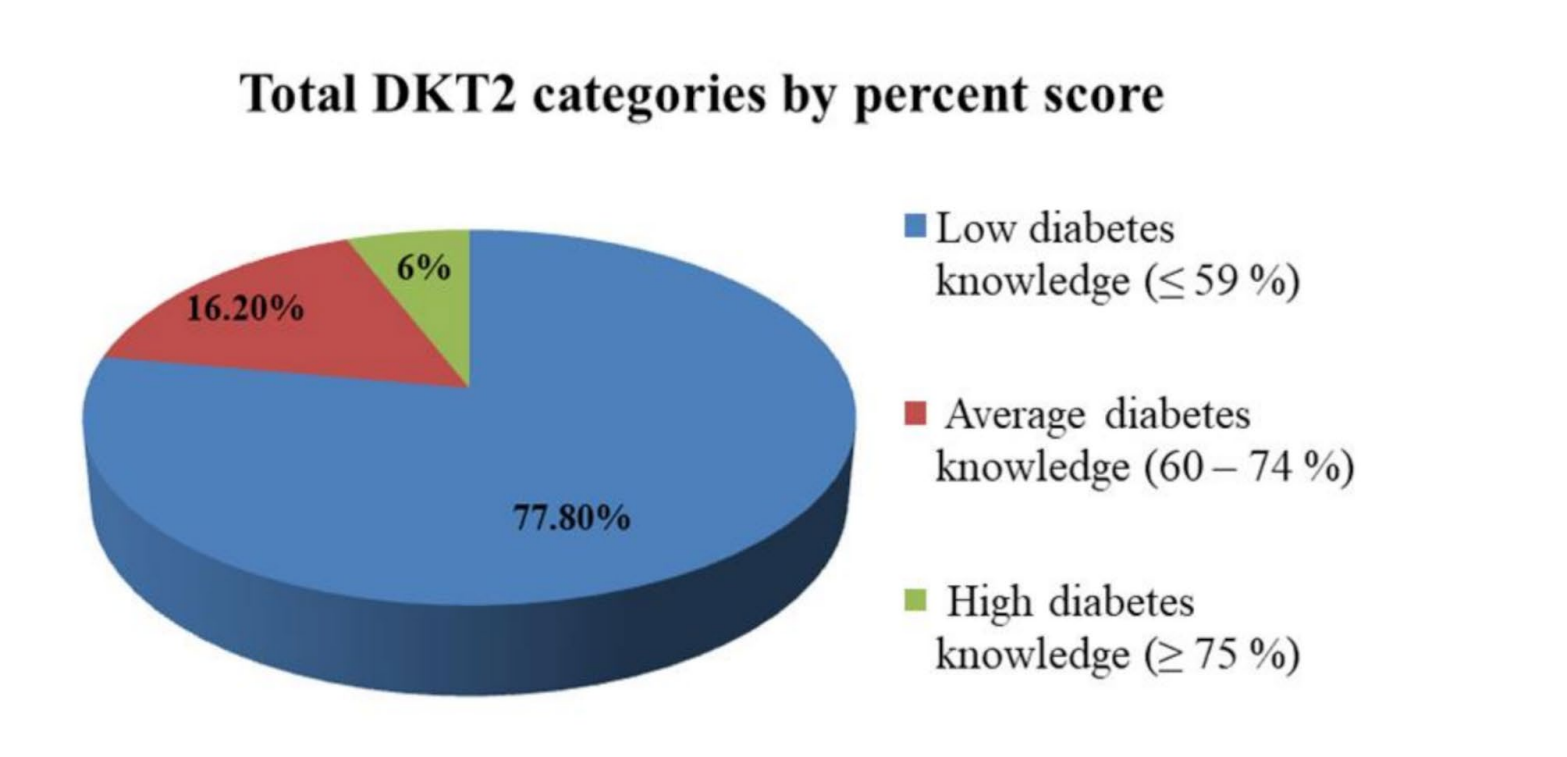
Levels of diabetes knowledge based on percent score of DKT2

The total score of the AAHLS ranged from 4 to 24 and the median score was 12.0. The score of part 1 of DKT2 ranged from 2 to 12 with a median score of 5.0 while the score of part 2 ranged from 3 to 7 with a median score of 4.0. The Total score of correct answers calculated by percentage ranged from 14.2 to 85.7% with a median percent score of 43.4%. The total score of STOFHLA range was 0–30 with a median score of 22.5 (Table 2). The highest correct answers were in understanding clinic appointment details (88.5%), understanding prescription instructions (75.2%) followed by understanding instructions on medication label (61.3%). The lowest correct answers were in understanding normal blood sugar ranges (32.3%) (Fig. 1). The median score of SDSCA was 2 (Table 2). Three quarters of the study participants (73.5%) had poor self-care activities (< 3) (Fig. 2) The total DKT2 score was calculated through percentage of correct answers for each participant, 77.8% had low diabetes knowledge (≤ 59%) and only 6% had high diabetes knowledge (≥ 75%) (Fig. 3).

**Table 2:** Mean, standard deviation (SD), and median of AAHLS, DKT2, STOFHLA, and SDSCA

Scales	Min. – Max.	Mean ± SD	Median	IQR
**AAHLS (** *n* ** = 400)**	4.0–24.0	12.89 ± 4.17	12.0	6
**DKT2**				
**Part 1 (***n*** = 400)**	2.0–12.0	6.43 ± 2.25	5.0	2
**Part 2 (***n*** = 129)**	3.0–7.0	4.72 ± 0.99	4.0	2
**Overall DKT2 Score (%) (** *n* ** = 400)**	14.2–85.7%	47.5 ± 14.8%	43.4%	17.3%
**STOFHLA (** *n* ** = 400)**	0.0–30.0	19.29 ± 8.86	22.5	3
**SDSCA (** *n* ** = 400)**	0.0-6.3	2.0 ± 1.1	2.0	1.4

AAHLS: All Aspects of Health Literacy Scale, DKT2: Diabetes Knowledge Test, SDSCA: Summary of Diabetes Self-Care Activities Scale, STOFHLA: Short Test of Functional Health Literacy in Adults

Association between health literacy and diabetes self-care activities was examined. A significant positive correlation was found between AAHLS and STOFHLA (*r* = 0.768), SDSCA (*r* = 0.338), DKT2 part 1 (*r* = 0.336) and DKT2 part 2 (*r* = 0.447). SDSCA was also found to have a significant positive correlation with both DKT2 part 1(*r* = 0.379), part 2 (*r* = 0.254) and STOFHLA (*r* = 0.338). Both AAHLS and SDSCA had significant negative correlation with HbA1c (*r* = -0.156 and -0.560 respectively) (Table 3**).**

**Table 3:** Correlation between AAHLS, SDSCA, DKT2 part 1 and 2, STOFHLA, and HbA1c

	AAHLS	SDSCA	DKT2 part 1	DKT2 part 2	STOFHLA	HbA1c
**AAHLS**		0.338*	0.336*	0.447*	0.768*	-0.156*
**SDSCA**			0.379*	0.254*	0.338*	-0.560*
**DKT2 part 1**				0.544*	0.396*	-0.384*
**DKT2 part 2**					0.472*	-0.186
**STOFHLA**						-0.189*
**HbA1c**						

* *p* < 0.001

AAHLS: All Aspects of Health Literacy Scale, DKT2: Diabetes Knowledge Test, HbA1c: Glycated hemoglobin, SDSCA: Summary of Diabetes Self-Care Activities Scale, STOFHLA: Short Test of Functional Health Literacy in Adults

Education, income level and practicing physical activities were found to have a statistically significant association with good self-care activities. For education, participants who had a university degree were found to have the higher odds of good self-care activities (OR = 1.941, 95%CI; 1.591–2.368, *p* < 0.001). As for income levels, participants who reported having enough income had higher self-care activities (OR = 1.941, 95%CI; 1.591–2.368, *p* < 0.001). Those who practiced regular physical activities were also found to have higher self-care activities (OR = 1.941, 95%CI; 1.591–2.368, *p* < 0.001) (Table 4**).** However, results of multiple logistic regression analysis shows that health literacy and diabetes knowledge were found to be the only significant predictors of good self-care activities ((aOR = 1.132; 95% CI:1.062–1.207, *p* < 0.001 and aOR = 1.313; 95% CI: 1.178–1.464, *p* < 0.001; respectively) (Table 5**).**

**Table 4:** Association between good diabetes self-care activities and general characteristics of the study sample

	Good diabetes self-care activities (*n* = 106)		Crude OR	LL	UL	*P* value
	No.	%				
**Age (years)**						
60–64	54	26.7	Ref.			
65–69	27	25.5	0.937	0.548	1.602	0.811
70+	25	27.2	1.023	0.587	1.782	0.937
**Gender**						
Male	56	24.9	Ref.			
Female	50	28.6	1.207	0.773	1.886	0.408
**Marital status**						
Unmarried	37	23.9	Ref.			
Married	69	28.2	1.250	0.787	1.985	0.344
**Education**						
Below university level	51	20.3	Ref.			
University graduate or above	55	36.9	2.295	1.459	3.609	< 0.001*
**Income**						
Not enough / In debt	31	17.9	Ref.			
Enough/ save	75	33.0	2.260	1.403	3.641	< 0.001*
**Occupation**						
Not working (housewife, retired)	99	28.0	2.163	0.936	4.997	0.071
Still working	7	15.2	Ref.			
**Living condition**						
Alone	20	30.8	Ref.			
Family	86	25.7	1.287	0.720	2.301	0.395
**Practicing regular physical activities**						
No	70	22.4	Ref.			
Yes	36	40.9	2.393	1.450	3.951	< 0.001*
**Current smoking**						
No	99	27.3	1.607	0.684	3.777	0.276
Yes	7	18.9	Ref.			
**Duration of diabetes (Years)**						
< 10	58	27.6	1.129	0.723	1.763	0.594
≥ 10	48	25.3	Ref.			
**Diabetes medications**						
Oral hypoglycemic	71	27.1	1.328	0.692	2.549	0.394
Insulin	21	28.4	1.415	0.649	3.083	0.382
Both	14	21.9	Ref.			

*: Statistically significant at *p* < 0.05

**Table 5:** Predictors of good diabetes self-care activities: results of multiple logistic regression analysis

Predictors of good diabetes self-care activities	B	SE	Adjusted OR	LL	UL	*P* value
**Constant**	-5.304	0.736	0.005			< 0.001*
**Health Literacy (AAHLS)**	0.124	0.033	1.132	1.062	1.207	< 0.001*
**Diabetes Knowledge (DKT part 1)**	0.273	0.055	1.313	1.178	1.464	< 0.001*

Backward LR Logistic regression analysis SE: Standard Error OR: Odds Ratio LL: Lower Limit UL: Upper Limit

Adjusted R^2^ = 0.260, X^2^ = 78.475, *p* = 0.000

*: Statistically significant at *p* < 0.05

All variables in the bivariate analysis with *p* value < 0.2 were entered into the model

## Discussion

The present study demonstrated that good self-care activities were independently associated with health literacy and diabetes knowledge. Moreover, diabetes self-care activities have a significant association with glycemic control. It was found that 73.5% of older adults with diabetes had poor self-care activities as measured by Summary of diabetes self-care activities (SDSCA) scale. Similar results (72.8%) were reported by Harikrishna et al. [25] in a community based cross-sectional study among older adults with diabetes in India. On the other hand, a study conducted in Ethiopia reported that only 49.1% of patients had poor self-care activities. However, this study was conducted among a younger age group (40–60 years old) [20].

Education was significantly associated with good self-care activities (SDSCA) in this study; those with university degrees had the highest self-care activities score. Most of the available literature suggests a strong significant association between level of education and HL [26]. Similarly another study in Egypt found that better diabetes knowledge was associated with higher level of education [27]. The consistency of these results is not surprising as knowledge is gained through education and adults with low education have been reported to have lower self-efficacy levels [20]. Furthermore, studies in Ethiopia and Iran reported that lower educational level was a significant predictor of poor self-care activities [28, 29]. Highly educated individuals have been found to have good decision-making abilities and self-efficacy specifically in context of diabetes self-care management. These results highlight the value of education in the promotion and attainment of HL and better self-care practices and good health outcomes in all individuals, especially the older adults.

Income level showed a statistically significant association with good self-care activities, with participants reporting sufficient income having higher self-care activity scores. Guo et al. [30], in their systematic review, also noted that health literacy was influenced by income level. Comparable findings were reported by Yılmazel & Cici [31] in Turkey and Xie et al. [32] in China. Additionally, a study in Iran [29] found a significant positive association between monthly income and diabetes self-care activities. People with higher income are more likely to be educated and mostly have better access to health information and health-care services through private channels. Furthermore, in our culture, people with poor income usually have more pressing priorities than focusing on their health. Diabetes self-care management also requires a certain level of income to maintain a healthy lifestyle, obtain a home glucose monitoring device and regularly follow-up with health-care professionals in absence of an adequate health insurance system. Therefore, it is imperative to assess income-related inequality in both HL and diabetes literacy, because rectifying this inequality is a potential opportunity to improve health outcomes in the population.

Physical activity had a statistically significant association with self-care activities in the present study. Older adults who practiced regular physical activity had higher scores on the SDSCA. A study in Brazil also found a statistically significant association between physical activity and better diabetes knowledge and positive attitude to self-care activities in older adults living with diabetes [33]. Vicente et al. [34] also reported a significant positive association between physical activity and some domains of self-care activities such as exercise and foot-care among older adults living with diabetes in Brazil. Patients who have good HL presumably know the significant value of physical activity in managing their condition and its impact on overall health status. Understanding the impact of physical activity on glycemic control in patients with diabetes is a crucial aspect of diabetes knowledge and self-care activities [35].

In the current study, HbA1c was found to have a significant strong negative association with self-care activities in older adults living with diabetes. Similar to the results in this study; a cross-sectional study in a university hospital in Korea reported that diabetes self-care activities had a significant negative correlation with both HbA1c and fasting blood sugar [36]. On the other hand, a study in Qatar reported no significant relationship between self-care activities and HbA1c levels [37]. This result was surprising because most of the available studies reported a significant relationship between them. Good self-care activities, among other factors, were found to cause better glycemic control and diabetes outcomes in several studies [38, 39].

HbA1c was also found to have a significant negative correlation with AAHLS, STOFHLA and DKT2. Similar to our study, studies in Turkey and Brazil reported that HL had a statistically significant negative association with HbA1c levels in older adults living with diabetes [31, 40]. Surprisingly, a cross-sectional study in Qatar, with a mean age of 50.7 years, reported that there was no significant association between diabetes knowledge and glycemic control [37]. In this context, low HL and diabetes knowledge prove to be vital variables that can explain the prevalence of poor glycemic control in patients with T2D. Several studies have highlighted the strong association between HL and diabetes management [38, 40, 41]. Sufficient HL was reported to be a necessity for the effective utilization of the tools of diabetes self-care management [42]. In the current study, a statistically significant correlation was found between all measures of HL and diabetes knowledge and self-care activities. Also, results of multiple logistic regression analysis shows that health literacy and diabetes knowledge were found to be the only significant predictors of good self-care activities. Moreover, Forghani et al. [43] in Iran and Zhao [44] in China reported a significant link between HL and diabetes self-care activities. Moreover, a cross-sectional study in Malaysia among older adults living with diabetes, reported a significant positive association between diabetes knowledge and self-care activities [45]. Several cross-sectional studies have also reported a positive association between diabetes knowledge and self-care activities [46, 47]. All these results highlight the significance of diabetes knowledge in achieving good self-care practice and eventually better glycemic control and disease outcome in older adults with diabetes.

The significant association between health literacy, diabetes knowledge, and self-care activities highlights the critical role of education and literacy in managing chronic conditions like diabetes. While our study was conducted in a specific population within Alexandria, the underlying principles regarding the impact of health literacy and diabetes knowledge on self-care are likely applicable to older adults’ population in Egypt, and also, in regions with similar sociodemographic profiles. However, caution should be exercised when generalizing these results to different cultural or healthcare contexts, where variations in healthcare access, education systems, and social support networks may influence the outcomes.

### Study limitations

One of the limitations of this study is related to the cross-sectional research design, which can’t verify the cause-effect relationships between the variables. Moreover, this study used subjective self-rated health questions which could be subject to various social implications. Also, the actual estimate of health literacy levels in the studied sample could not be assessed or compared to other studies because all aspects of health literacy scale (AAHLS) have no cut-off score or universal total score.

### Conclusion and recommendations

Health literacy and diabetes knowledge were identified as predictors of good self-care activities in older adults with diabetes, and all three; health literacy, diabetes knowledge, and self-care activities were significantly associated with diabetes outcomes as measured by HbA1c. Based on these findings several recommendations can be made. Firstly, enhancing health literacy programs for older adults with type 2 diabetes is crucial. These programs should be tailored, particularly for individuals with lower educational backgrounds, to improve their understanding and practice of diabetes management, medication adherence, and lifestyle modifications. Additionally, it is recommended to develop and offer personalized diabetes education sessions that cater to the individual needs of older adults. These sessions should consider the varying levels of health literacy and diabetes knowledge among patients to ensure effective learning and application of self-care practices. Furthermore, incorporating routine health literacy assessments into clinical practice can help identify those with low health literacy early, allowing healthcare providers to offer additional support and resources. By focusing on these areas, healthcare professionals can better support older adults in managing their diabetes, ultimately improving self-care activities and glycemic control.

## Data Availability

The data that support the findings of this study are available from the corresponding author upon reasonable request.

## Abbreviations

AAHLS: All Aspects of Health Literacy Scale
ADA: American Diabetes Association
aOR: adjusted odds ratio
BMI: Body Mass Index
DKT2: Diabetes Knowledge Test
HL: Health Literacy
HbA1c: Glycated hemoglobin
SDSCA: Summary of Diabetes Self-Care Activities Scale
STOFHLA: Short Test of Functional Health Literacy in Adults
USA: United States of America
